# Brain computer interfaces for cognitive enhancement in older people - challenges and applications: a systematic review

**DOI:** 10.1186/s12877-025-05676-4

**Published:** 2025-01-16

**Authors:** Ping-Chen Tsai, Asangaedem Akpan, Kea-Tiong Tang, Heba Lakany

**Affiliations:** 1 Department of Electronic and Electrical Engineering, University of Liverpool, 9 Brownlow Hill, Liverpool, UK; 2 Department of Electrical Engineering, National Tsinghua University, Hsinchu, Taiwan; 3 Institute of Life Course & Medical Sciences, University of Liverpool and Liverpool University Hospitals NHS FT, Liverpool, UK; 4 NIHR Clinical Research Network, Northwest Coast, Liverpool Science Park, Liverpool, UK; 5 Present Address: Division of Internal Medicine, University of Western Australia, Nedlands, Western Australia Australia

**Keywords:** Brain computer interface, Neurofeedback, EEG, Cognitive performance, Older people, Healthy, Mild cognitive impairment

## Abstract

**Background:**

Brain-computer interface (BCI) offers promising solutions to cognitive enhancement in older people. Despite the clear progress received, there is limited evidence of BCI implementation for rehabilitation. This systematic review addresses BCI applications and challenges in the standard practice of EEG-based neurofeedback (NF) training in healthy older people or older people with mild cognitive impairment (MCI).

**Methods:**

Articles were searched via MEDLINE, PubMed, SCOPUS, SpringerLink, and Web of Science. 16 studies between 1st January 2010 to 1st November 2024 are included after screening using PRISMA. The risk of bias, system design, and neurofeedback protocols are reviewed.

**Results:**

The successful BCI applications in NF trials in older people were biased by the randomisation process and outcome measurement. Although the studies demonstrate promising results in effectiveness of research-grade BCI for cognitive enhancement in older people, it is premature to make definitive claims about widespread BCI usability and applicability.

**Significance:**

This review highlights the common issues in the field of EEG-based BCI for older people. Future BCI research could focus on trial design and BCI performance gaps between the old and the young to develop a robust BCI system that compensates for age-related declines in cognitive and motor functions.

## Introduction

Age-related changes in memory and brain function decline lead to increased dependency in older people. Changes in brain cognition can be profound as the individual reaches middle age and decline further as people aged [1]. Compared to younger individuals, older people tend to have a more difficult time remembering and learning new skills. Deficiencies in motor skill learning can result in an inability to climb stairs, maintain balance, or drive safely due to deficiencies in muscle and skeleton movement. The older people have to contend with not only cognitive and physical impairments, but funding their care and treatment which can be expensive.

In the last decade, brain-computer interfaces (BCI) has become a growing field of research [2]. BCI is a technology that enables direct communication between the brain and an external device, often used to assist, augment, or repair human cognitive or sensory-motor functions. The increasing use of personal computers by older people has led to the development and application of BCI for their well-being [3]. Many researchers have been studying the application of BCI in neuronal rehabilitation for healthy older people and older people with mild cognitive impairment (MCI). BCI helps cognitive enhancement in older brains and age-related changes in brain dynamics. One of the most common applications of neuronal rehabilitation is based on the method of non-invasive neurofeedback (NF).

**Fig. 1:**
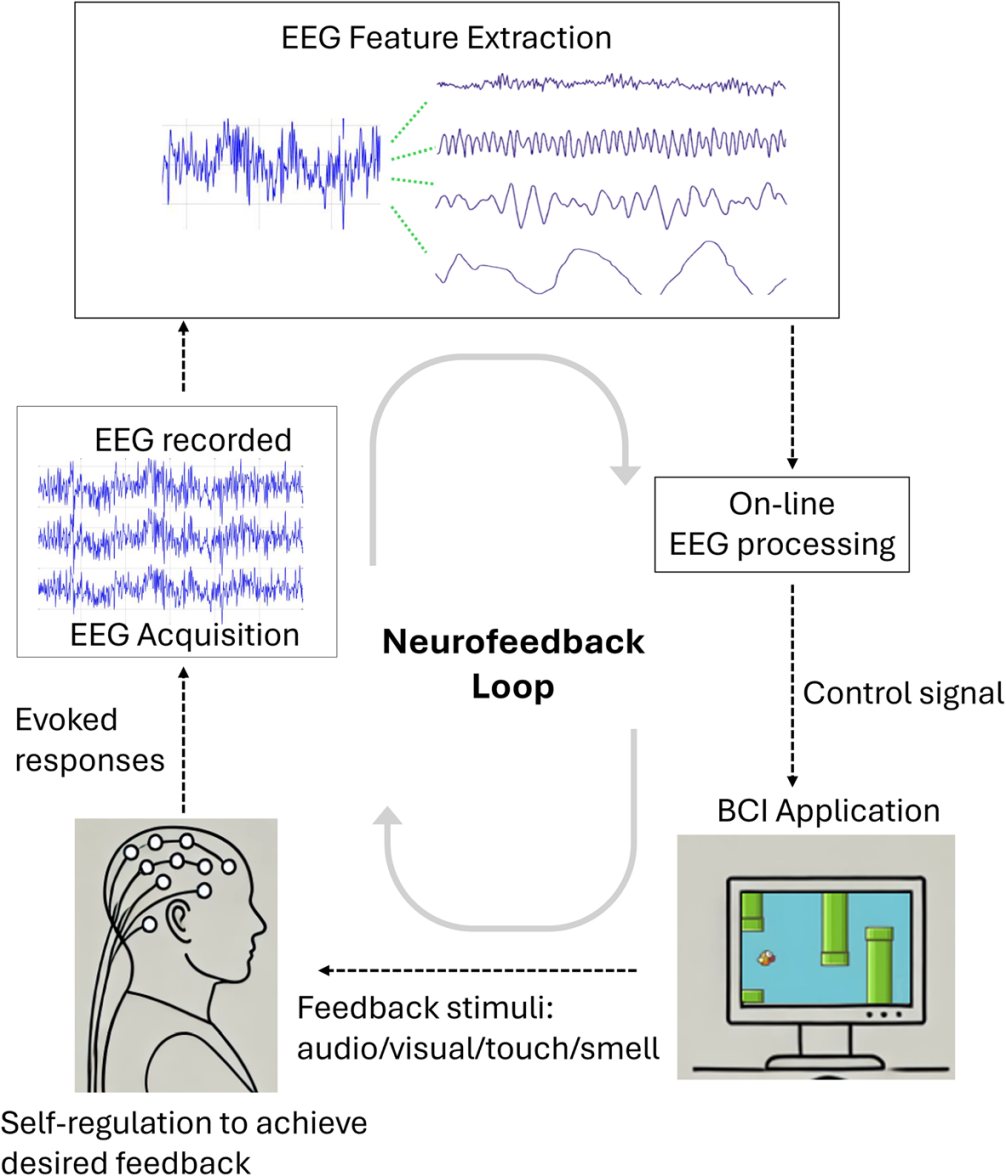
Neurofeedback Loop - EEG sensors record brain activity, which is then processed and analysed. Control commands are generated and feedback stimuli, such as visual game elements, are provided to the subject, completing the neurofeedback loop

Neurofeedback (NF) is a biofeedback technique that uses real-time brain activity displays to teach self-regulation, based on Hebbian Plasticity and Operant Conditioning [4]. Hebbian Plasticity explains how neurons adapt during learning and memory formation through synaptic strengthening when activated together [5, 6]. Operant Conditioning, developed by B.F. Skinner [7], involves learning through rewards and punishments. In NF, individuals receive positive reinforcement when their brain activity aligns with desired states, promoting self-regulation and beneficial brain states. During EEG-based NF interventions, subjects receive stimuli from a BCI application. They then perform self-regulation of brain activity. Changes in brain activity are observed through EEG alterations, representing evoked responses. EEG data is recorded and features are extracted for analysis. These features are then processed to generate control commands, which are sent back to the interface to provide feedback stimuli. Feedback stimuli can be auditory, visual, haptic, or olfactory. This completes the neurofeedback loop as shown in Fig. 1.

Neuronal activities are produced by the activities of cerebral neurons represented by electrical pulses. The synchronous activity of electrical pulses reflects the pattern of brain waves. Electroencephalography (EEG) represents the brain activities and is recorded during NF. The frequency components of EEG are commonly classified into delta (less than 4 Hz), theta (4–7 Hz), alpha (8–14 Hz), beta (15–30 Hz), and gamma (31–80 Hz). Each band represents the characteristics of different physiological functions. Delta activity is more related to sleep and deep unconsciousness [8]. Theta power is a salient index of working memory and spatial memory [9]. Healthy older people show a decrease in alpha power in frontal and parietal areas at rest. This phenomenon could be a reflection of impaired attention [10]. Previous investigations into how EEG signals change in healthy older people suggested that alpha amplitude attenuation is indicative of cognitive decline. This leads to poorer performance in memory load tasks compared to young people [11]. Beta wave is more prominent when a person is awake and paying attention [12]. Gamma activity is observed when the person is learning and problem-solving [13] (Table 1). These frequency components are used as feedback biomarkers throughout the NF trials to train particular cognitive functions. NF trials have produced consistent findings suggesting that the BCI system is robust [14].

**Table 1:** An overview of the physiological processes associated with different frequency bands, the corresponding cortical areas, and describes how changes in brain activity due to aging are reflected in these bands

Frequency band	Physiological process and changes in normal adults	Cortical area	Changes due to aging
Delta (0.5–3 Hz)	Deep sleep, unconscious processes (increase in amplitude), increased awareness (decrease in amplitude) [8]	Frontal lobes, thalamus [15]	Decrease in amplitude, reflecting reduced slow-wave sleep [16]
Theta (4–7 Hz)	Learning, memory, increased cognitive load, internal focus (increase in amplitude), drowsiness, calmness (decrease in amplitude)	Hippocampus, frontal lobes [17]	Potential increase, associated with age-related cognitive decline [18]
Lower Alpha (8–10 Hz)	Relaxed wakefulness, closed eyes, recalling (increase in amplitude), reduced relaxation (decrease in amplitude)	Occipital lobe	Decrease in amplitude and frequency, indicating reduced relaxation and attentiveness [19]
Upper Alpha (10–14 Hz)	inhibitory processes, alertness (increase in amplitude), semantic memory (decrease in amplitude)	Frontal, temporal, parietal lobes [10, 20]	Decrease in amplitude and frequency, indicating reduced cognitive efficiency [11]
Beta (15–30 Hz)	Related to sensorimotor processing. Focused execution and controlling (increase in amplitude), uncertainty about how to act (decrease in amplitude) [12]	Frontal, parietal, occipital lobes	Decrease in amplitude, reflecting changes in cognitive processing [21, 22]
Gamma (31–80 Hz)	High-level information processing(increase in amplitude), reduced sensory drive (decrease in amplitude)	Sensory cortex at frontal and parietal lobes [13]	Reduction in power, associated with decreased cognitive performance [23, 24]

Of note, using BCI to enhance cognitive functions for general population on the NF has been widely trialled to investigate the effectiveness on improvement of neurological conditions such as motor paralysis after stroke [25, 26], spinal cord injury [27] or ADHD [28]. Because ageing can significantly affect brain function, it is important to study the impacts of healthy and MCI-related differences in EEG when designing BCI systems for older people to ensure their effectiveness. The method of NF to treat conditions in older people has been reviewed [29–31]. The design of BCI systems, however, has not been discussed in these reviews. The common protocols of EEG-based NF have been assessed and the applied BCI system designs in individuals, including but not limited to older people, have been briefly elucidated [32]. In the past, challenges of BCI regarding ethical issues [33, 34], signal classification [35, 36], user interaction [37] and industrial performance [38] have been reviewed. Most reviews, however, do not address contemporary trends and limitations of BCI system design applied particularly to the older population. The growing older population and evidence that NF benefits older people necessitate a review to assess objectively the applications of BCI and the potential challenges [32, 38]. With the development of electroencephalogram (EEG) technology, BCI applications for NF prevention of older people cognitive declines have also been developed to improve the quality of life of older people. The need for validated and verified NF trials conducted on healthy older people and older people with mild cognitive impairment (MCI) becomes increasingly important as the older population accounts for 12% of the population globally and will increase to 21% by 2050 [39].

A key recommendation of cognitive decline literature and the development of NF observed in recent years is a thorough review of BCI applications. Here we conduct a systematic review in the light of the latest studies to explore the following aspects of BCI application: risk of bias of the NF trials, BCI system design, EEG processing procedure, NF paradigm and NF mechanism. We also discuss technical and clinical challenges of BCI when implementing NF on healthy older people and older people with MCI for the purpose of cognitive enhancement. Having extensive research studies [24, 28, 32, 40–43] reporting the benefits brought to the society by NF training, the necessity to conduct a review based on EEG-based NF methods in older people is suggested.

## Method

The present review summarises the last 14 years (2010–2024) of research on BCI NF methods to improve cognitive function, addressing the following two questions: (1) How does BCI enhance the cognitive performance by using NF method among healthy older people or older people with mild cognitive impairment? (2) What are the challenges observed in these applications?

The review protocol is registered with the Open Science Framework (OSF): https://osf.io/t69hd [44].

### Eligibility criteria

This is a systematic review (SLR).The inclusion and exclusion criteria are defined by the research question and patient/population, intervention, comparison and outcomes (PICO) format. Eligible source articles are published in English in journals if the sources were focused on the brain computer interface, NF training, and cognitive functions improvement in older people.

The inclusion criteria are: 1. Study design types: intervention studies. 2. Participants: healthy people, people with MCI (subjective to screening criterion in the studies). Age is limited to 55+ years. 3. brain imaging types: electroencephalogram (EEG) 4. Outcome measures for data: studies were included if they had at least a standardised test for the corresponding cognitive functions; if the cognitive capability, learning, attention, executive function, memory, processing speed or other cognitive functions were assessed; if their results were based on analysis of the participant’s neurological assessment scores or their EEG data. 5. Studies using original primary data.

The exclusion criteria are: 1. Any review papers and meta-analysis, letters, notes. 2. Any articles that did not meet inclusion criteria or did not include outcome measures related to the inclusion criteria. 3. Brain imaging type: MRI fMRI, transcranial magnetic stimulation (TMS) 4. Participants: Adults with the following conditions: severe dementia, severe neurological diseases and psychiatric disorders (anxiety or depression). People who are inpatient or stay in acute care are excluded. 5. Studies that lack reporting of (a) evidence such as the selected frequencies or location of electrodes (b) use of EEG signals in NF training.

### Search strategy

**Sources:** We searched for all BCI application on NF studies conducted from 1st January 2010 up to 01 Nov 2024 via MEDLINE, PubMed, SpringerLink, SCOPUS, and Web of Science.

For the search filter applied for the database that enables Boolean operators, we applied the following query for reviewing cognitive function improvement in older people using NF method:

( NF OR biofeedback OR “brain computer interface” OR “brain modulation” OR brain OR bci ) AND ( home OR community OR resident* OR “care home” OR geriatric OR “care unit” ) AND ( eeg OR electroencephalography OR motor AND imagery OR sensorimotor OR potential OR cortical OR “neural activity” ) AND ( elder* OR elderly OR “older People” OR aging OR ageing OR senior* ) AND ( “Cognitive function” OR “Cognitive deficit*” OR “Cognitive aging” OR “cognitive improve*” OR “cognitive training” OR “mild cognitive impair*” OR memory OR attention OR executive OR “processing speed” ) AND NOT ( review OR meta OR “meta analysis” OR guide*)

Additional filters were applied to fit the eligibility criteria.

## Study selection

Articles on BCI for training older people’s cognitive capability are in journals and conferences of various disciplines. They could be found in both engineering and non-engineering journals, including Frontiers in Aging Neuroscience, Neurobiology of Aging, Human Brain Mapping etc.

A screening process was conducted. The screening sequence is as follows: title reading, abstract reading, and full text reading. Those not meeting the eligibility criteria were rejected.

## Data collection process

**Fig. 2:**
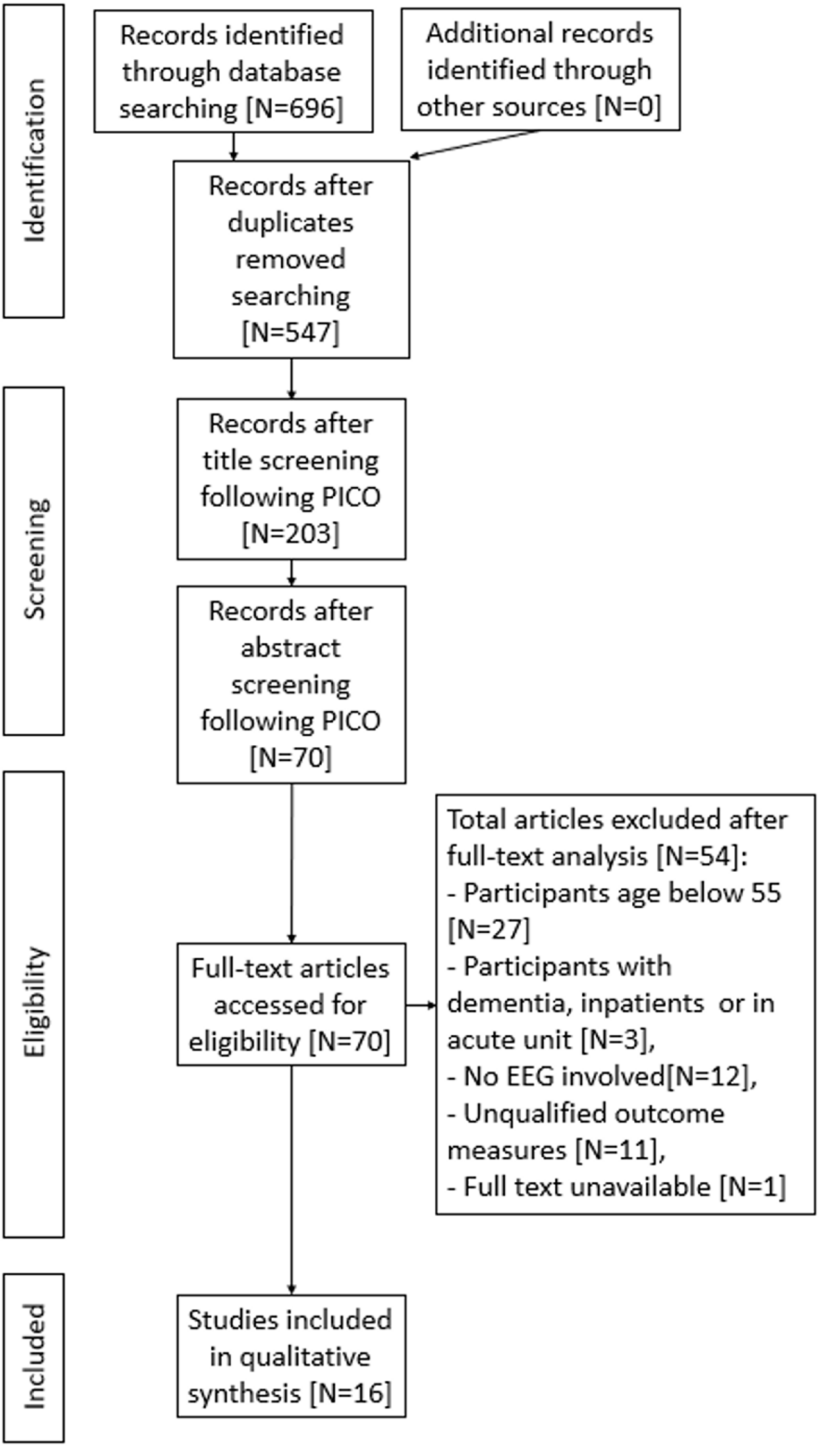
The PRISMA flowchart depicts the phases of this systematic review. The number of included and excluded studies are recorded in the chart

This systematic review follows the Preferred Reporting Items for Systematic Reviews and Meta-Analyses (PRISMA) [45]. A flowchart for article screening and data selection was presented in Fig. 2. The extraction of articles uses predefined keywords in “Search strategy” section. The search process initially identified 696 records via MEDLINE, PubMed, IEEE xplore, Scopus, SpringerLink,and Web of Science. Duplicated records were removed and 547 unique records remained. Through title screening and abstract screening following PICO, 70 records met the inclusion criteria and were retained. After full-text examination, 16 studies were included in this review.

### Data extraction

The following data was extracted from the eligible studies: (1) Risk of bias (2) Hardware and software used for NF training (3) Feedback electrode placement (4) NF training methods (5) EEG processing (6) Impact on cognitive performance due to the intervention.

## Synthesis of results

The synthesis of results pools data from studies at all levels of bias, including high risk of bias, since it is crucial to know the details of each study. Examination of all the included trial studies can reveal what aspects of trial design and outcomes are likely to be accepted at a ’low’ risk of bias. A narrative approach is used to synthesise the results.

### System design

While three of the included studies did not include pre-post EEG analysis results [46–48], the rest of the studies showed the effect of NF through the analysis of recorded EEG. The EEG caps used for EEG recording have a number of active electrodes from 2 [49] to 32 [50]. However, not all active electrode positions were used for feedback.

The two popular research-grade EEG recording equipment for BCI are the 10-channel amplifier, NeXus-10 MKII by Mind Media [51–53], and amplifiers by Brain Product [52–54]. Five studies separated the amplifiers for resting EEG recording and NF training feedback EEG recording [50, 52, 53, 55, 56]. The NF paradigm is an important component of the experiment design. The user friendliness of the paradigm is essential to ensure the acceptance by the participants. From the included studies, the NF paradigms were generated either by customised BCI systems [49, 54, 57, 58] or by general-purpose platforms [46–48, 50–53, 55, 56, 59–61]. Among the studies reviewed, BioGraph Infiniti [47, 50, 56], BCI2000 [59] and BioTrace+ (Mind Media) [51, 53] were the top three general-purpose BCI platforms used. Brain Vision Analyzer was the most popular off-line signal processing software in this field. Details are tabulated in Table 6 in Appendix.

The use of personal computers among older adults gives rise to the possibility of a home-based cognitive training programme. However, among the 16 included studies, 15 of them used research-level EEG headsets and amplifiers which were preferably kept and used under the supervision of technical staff in the laboratory. One exception from the included studies by Reichert et al. [52] conducted NF training in a retired stroke patient’s home. The stroke patient was identified as the target user of the NF training system and his EEG data was compared with healthy controls. Jirayu et al. [46]’s study used a commercial EEG headset, EMOTIV EPOC, for the NF training experiment. The headset consists of a 14-channel cap and a built-in signal acquisition system. The study by Sanchez-Cifo et al. [48] is the second included study which deployed a commercial device, Muse 2 headset, for the training. The device has 2 electrodes and is highly portable that is suitable for a broader audience like older adults. The headset was paired with a Polar OH1, an optical heart rate monitor that uses Photoplethysmography (PPG) technology to measure heart rate, to provide complementary physiological data.

All included studies focused on validating proof of concept among older adults by implementing a NF protocol. A challenge is that there is a lack of usability and user acceptance measure of research-grade EEG device in the included literature. This is due to the rigour in collecting and filtering the most relevant outcome data for answering the research questions. Participants’ reactions to the BCI system reflect their engagement and comfort with its use. When using BCI for intervention, the researcher will have to think about how comfortable the system is, or how rewarding the stimuli are to them. A rating scale or qualitative approach can be employed to measure user satisfaction and hence direct future BCI system design for older adults [62].

### User interface

All the included studies applied a graphical interface for NF training that provided visual feedback to the users. The cognitive functions components (e.g. attention, working memory, visual processing etc.) were measured either through interaction tasks’ response time or frequency band power values. In six of the studies, the stimuli used enabled decomposition of cognitive process into various cognitive functions [46, 49, 52, 54, 56, 59]. That is, a cognitive task was used to train the individual components. The interaction between the user and the computer could be quantified by the difficulty of cognitive tasks in the real-time graphical interface. The interaction tasks in the included studies include recalling game objects and modulating brain activities to control game characters. “NF training methods” section will elaborate on the interaction tasks designed by the included studies in this systematic review.

Many of the excluded studies [63–68] also used task decomposition to approach cognitive functions training without the help of EEG signals. Instead, they used game pads, consoles such as Nintendo Switch^®^, Wii^®^, Oculus^®^ quest and traditional mouse and keyboard for interactions. These commercially available gaming devices create the possibility of more effective interfaces. On the other hand, research-grade BCI systems can provide the precision and accuracy that the excluded studies cannot achieve. Research has shown that maintaining high accuracy in BCIs is essential for effective neurofeedback training [69]. It is essential to maintain the precision and accuracy of BCI systems while integrating various neurofeedback paradigms for more effective interfaces.

### Feedback electrode placement

All the included studies employed the International 10–20 system of electrode placement which standardises areas of the skull and facilitates EEG data comparison. Generally, the NF feedback electrode(s) are placed in a way that the trained frequency at the position is associated with the cognitive functions aiming to improve.

One prevalent approach to NF training is EEG spectrum regulation training at a particular brain region. Each region is responsible for specific tasks or feelings. For example, the sensory and motor (sensorimotor) cortex with montages Cz, C3 and C4 are related to functions of movement control and the ability to recognise body sensations. Another prominent EEG oscillation is in the frontal lobes: Fp1, Fp2, Fpz, Fz, F3, F4 and F7, which are related to immediate and sustained attention, and selective attention [70, 71]. Therefore, it is critical to identify these areas and determine a reasonable NF strategy.

The studies that pursued SMR NF training commonly placed the feedback electrode in the central region. Training for alertness and attention was conducted at the frontal and prefrontal sites. Short-term memory and information processing improvement training involves control of the individual band at central sites. Training to improve episodic memory retrieval normally places the feedback electrode at parietal sites. Depending on the outcome measure, electrode positions may be different between studies for the same training frequency band. The placement and frequency of interest for each study are summarised in Table 2.

**Table 2:** The table shows the selected frequency bands for NF training

Reference	Reward EEG component(s)	Feedback electrode placement
Alatorre et al. [61]	Z value of theta (4–7.5Hz) AP^a^	Lead with the most abnormal AP
Andrade et al. [58]	Gamma (30-50Hz)	Average phase synchrony of Fz, Cz, Pz, C3, and C4
Azarpaikan and Torbati [47]	SMR (12-15Hz) and Theta (4-7Hz)	O1, O2
Becerra et al. [60]	Z value of theta (3.6–7.5Hz) AP	Lead with the most abnormal AP
Campos et al. [56]	SMR (12–15 Hz)	Cz
Gomez et al. [59]	Alpha (12 ± 3Hz,) and beta (18 ± 3Hz, 21 ± 3Hz)	C3, Cz, C4
Jirayu et al. [46]	Beta (12–32 Hz)/ alpha (8–12 Hz)	Frontal lobe: AF3, AF4. Occipital lobe: O1, O2
Kober et al. [53]	Upper alpha (10–12 Hz)	Pz
Kober et al. [51]	SMR (12–15 Hz) and upper alpha (Individual alpha frequency + 2 Hz)	SMR over Cz. Upper alpha over Pz
Lee et al. [49]	No chosen frequency bands	Pre-frontal cortex: Fp1, Fp2
Marlats et al. [55]	SMR (12–15 Hz), theta (4–7 Hz) and upper beta (13–20 Hz)	Cz
Reichert et al. [52]	SMR (12–15 Hz)	Cz
Reis et al. [54]	Alpha (IAPF^b^ ± 2 Hz) / baseline PSD, and theta (4 Hz to IAPF - 3 Hz)/ baseline PSD	Fz
Sanchez-Cifo et al. [48]	No chosen frequency bands	AF7, AF8
Staufenbiel et al. [57]	Beta (12–20 Hz) and gamma (36–44 Hz)	Fz
Wang and Hsieh [50]	Theta (4–7 Hz), 0.5–2 Hz (inhibited) and 43–49 Hz (inhibited)	Fz

^a^*AP* Absolute Power

^b ^*IAPF* Individual Alpha Peak Frequency

In the majority of the 16 included studies, alpha band power is used as a focus for primary cognitive improvement training, as the suppression of alpha power is associated with the inhibition of sensory information. Different from other included studies, Alatorre et al. [61] and Becerra et al. [60] selected the lead with the most abnormal value of theta power among the other 19 leads. Jirayu et al. [46] chose placement AF3 as it is associated with attention state. To adapt to the symmetric design of the headset used, position AF4 was also selected for feedback. As proposed by the author [46], the EEG signals from this lead provided feedback signals for the training when the theta power exceeded or fell below the target threshold. Locations at the occipital lobe, O1, and O2, were more suitable for artefact removal purposes because they are further away from the human face compared to the locations at the frontal and temporal regions. Another noteworthy feedback approach is that of Andrade et al. [58], which employed average gamma-band phase synchrony (30–50 Hz) in the fronto-central and parieto-central regions (Fz, Cz, Pz, C3, and C4) as feedback signal. When designing the experiment, the researcher should first examine the cognitive function to be trained before choosing suitable feedback electrodes.

### NF training methods

BCI control correlates with cortical activity intensity. The duration of NF sessions is essential in an experimental protocol for older people because they find it difficult to stay awake when sitting still for a long time. However, an insufficient amount of NF training time leads to the ineffectiveness of the training. Table 3 lists the number of training sessions and session duration times for the included studies, with durations spanning from 30 to 90 minutes. Only one study [48] did not specify the number of training sessions or the session duration. Important factors for the BCI system design specific to the study are the preparation time, the strength of the signal of interest, EEG recording quality, comfort for the participant, robustness to motion artefacts, and flexibility for multiple head sizes. Because the included published works considered a smaller phenomenon that requires high signal quality to be detectable, research-grade system are more robust to noise and thus became more suitable for complex analysis goals (Table 6 in Appendix). There were two main designs for EEG-based NF training: the motor imagery (MI)[72, 73] based method and the frequency-based method [32].

**Table 3:** The table gives out session duration and total number of training sessions

Reference	Duration(min)	Session No.
Alatorre et al. [61]	30	30
Andrade et al. [58]	30	20
Azarpaikan and Torbati [47]	30	15
Becerra et al. [60]	30	30
Campos et al. [56]	30	10
Gomez et al. [59]	90	5
Jirayu et al. [46]	30	20
Kober et al. [53]	45	10
Kober et al. [51]	45	10
Lee et al. [49]	30	24
Marlats et al. [55]	75	20
Reichert et al. [52]	50	10
Reis et al. [54]	30	8
Sanchez-Cifo et al. [48]	/	/
Staufenbiel et al. [57]	30	8
Wang and Hsieh [50]	Not reported	12

#### Motor imagery (MI)

MI-based NF training translates event-related desynchronisation (ERD) and event-related synchronisation (ERS) generated during motor imagery tasks into control commands. MI-based NF training specifically distinguishes between left and right motor imagery, with cortical activation patterns reflecting this distinction. For example, motor planning and execution typically exhibit left-lateralised cortical activation in right-handers but right-lateralised in left-handers [74]. This provides more complex feedback related to motor tasks. When conducting a motor imagery task, the participants perform the movement with all the sensory information to produce imagined motor control [72]. There is one included studies which employed an MI-based training protocol [59]. The MI-BCI game required the player to improve their motor imagery skills over time to meet the increasing demands of controlling the cursor. The participants perform five right and left MI tasks such as overcoming obstacles and memory exercises. Each of the five sessions has its own MI NF activities that instruct the participant to learn and adapt their MI strategy. MI-based neurofeedback training involves participants imagining four key stages of motor execution: goal selection, high-level planning, plan encoding, and plan execution [75, 76]. Performing MI can be more challenging and mentally demanding compared to frequency-based neurofeedback. For frequency-based NF, participants typically regulate their brainwave frequencies through relaxation or focused attention. Studies have shown that MI-based tasks often require extensive training periods for participants to achieve proficiency in controlling the neurofeedback interface (e.g., cursor movement) due to the complexity of motor imagery tasks [77, 78].

#### Frequency training

Although both motor imagery (MI)-based and frequency-based neurofeedback (NF) training aim to modulate brain activity, they differ fundamentally in their approaches. In contrast to MI, frequency-based NF training focuses on modulating particular EEG frequency bands to enhance cognitive functions, without differentiating between hemispheric activities. The training quantifies EEG frequency bands’ power and visualises the power in real-time for the participants to see. The powers can be visualised as a processing bar, the jumping height of an avatar, the speed of a displayed object, etc.

An entertaining audio-visual feedback in the NF training proposed by Wang and Hsieh [50] was provided in the form of a roller-coaster animation. There were five blocks of roller-coaster game in each session, where each block required the participant to increase the frontal midline theta power (4-7Hz). The amplitude of the frequency band was represented by a roller coaster speed. Likewise, Andrade et al. [58] used a growing tree animation to visualise neuromodulation. If the real-time computed value of the targeted biomarker (average Gamma-band phase synchrony) exceeded a pre-computed threshold, the tree would grow taller or become more vibrant. Lee et al.’s protocol [49] used a colour-word matching task to measure the participant’s baseline attention level. The participants then were required to maintain the attention level for two seconds to flip the cards over in a card-matching memory task. The protocol proposed by Jirayu et al. [46] also converted the frequency activities to an attentional level, where the attention level threshold for feedback was acquired in a calibration sessions before each training. The participant was expected to control the game object, e.g. car speed, using the attentional level. There were upper and lower attention thresholds for the system, and these could be adjusted to fit individual gaming capabilities.

During NF training, eye blinks and muscle movements can interfere with the modulation of frequency band power. The artefacts could be removed via an artefacts rejection scheme implemented after EEG data sampling (see Table 6 in Appendix and “EEG processing” section for on-line data processing details). There have been attempts to apply suppressing muscle artefacts as part of the EEG modulation strategy for the users to achieve training task goals. The modulation criteria are listed in Table 4. Seven studies included muscle artefact suppression as one of the feedback conditions that require more neural activity control from the session participants.

A prevalent feedback strategy was to visualise the real-time power values into bars [51–53, 56]. The height of the bar in the middle represents the frequency being trained, and the bar values at two sides represent the inhibited frequency values produced by the eyeblinks and muscle movement artefacts. Two studies designed the middle bar to reflect SMR (12-15Hz) power [51, 52]. Two studies designed the middle bar to reflect upper alpha power (UA) [51, 53]. Kober et al. [51] reported that 70$\%$ of the intervention group stroke participants were able to increase the NF feedback frequency power over the training runs, and 79$\%$ of the healthy participants in the control groups were able to increase the power of the feedback frequency.

Using a bar level to reflect spectrum power can be extended to a movie-watching task designed by Marlats et al. [55]. The task was to adjust the colour contrast and sound articulation in a historical reportage by increasing the SMR power and suppressing theta and upper beta power. This experiment design is similar to the author’s previous work, where the experimental design used the continuous feedback of the SMR/delta ratio and the upper beta/theta ratio to adjust the reportage quality [79].

Unlike other studies that utilised extensive electrode placements and targeted cognitive improvements, Sanchez-Cifo et al. [48] prioritised emotional regulation training with minimal equipment. They employed two electrodes at AF7 and AF8 to capture EEG and PPG signals for classifying participants’ emotional states (calm/stressed) using a supervised model in real-time. The model was trained to learn frequency bands linked to cognitive and emotional processes to distinguish between stress and calm states effectively. Stress levels were displayed on a visual scale to provide participants with practical and portable feedback for emotional regulation training.

For BCI, the transfer rate of signals continuously adjusts to the user’s EEG signal. A quick and precise operation is required. The technical challenge to date is the slow EEG transfer rate in BCI. Moreover, the information transfer rate may deteriorate in older people which might lead to a performance gap caused by older age [80]. Furthermore, it is not certain that an older human subject will interpret the feedback signal as a reward. The psychophysiological and neurological challenges are the emotional and mental mechanisms that can result in intra- and inter-individual heterogeneity. BCI users’ specific social demographics such as friendship network and age also play vital roles in brain dynamics that might affect reinforcement learning through NF [81].

**Table 4:** The table gives out the NF EEG modulation conditions for users

Reference	Muscle artefacts inhibition?	Reinforced power feedback conditions
Alatorre et al. [61]	/^a^	Theta should be in between 60–80$\%$ of previous power
Andrade et al. [58]	/	Gamma synchrony up-regulation
Azarpaikan and Torbati [47]	/	Theta power suppression and SMR power up-regulation
Becerra et al. [60]	/	Theta should be in between 60–80$\%$ of previous power
Campos et al. [56]	Power bands related to motor was not controlled	SMR power in a 1-s window increases by 10$\%$ for each baseline measured
Gomez et al. [59]	/	Modulation around 12Hz, 18Hz and 21 Hz
Jirayu et al. [46]	/	Beta/Alpha ratio exceeds the average of current value and its three previous epochs
Kober et al. [53]	Keep theta and beta below a certain threshold (eye blinks and movements)	Upper alpha power surpass mean power of baseline and previous run
Kober et al. [51]	Suppress power band 0.05–10 Hz (eye blinks) and 75–100 Hz (Muscle artefacts)	SMR and upper alpha power surpasses mean power value of the baseline run and previous feedback run
Lee et al. [49]	/	Attentive score above 50 lasting for 2 seconds. (No specific frequency band)
Marlats et al. [55]	Suppress beta band power	SMR and theta reach 50$\%$ of the maximum amplitude of baseline measurement
Reichert et al. [52]	Suppress eye-blink artefacts (4-7Hz band power)	SMR power exceeds baseline mean +1 standard deviation for more than 250 ms
Reis et al. [54]	Suppress Fp1 or Fp2-Fp1 signal amplitude brought by eye movements	Real-time ratio of alpha or theta PSD to baseline PSD was visualised
Sanchez-Cifo et al. [48]	Muse 2 built-in preprocessing algorithm	calm/stressed states
Staufenbiel et al. [57]	/	Beta and Gamma power level surpass at 75$\%$ of the power in the preceding 30 sec window
Wang and Hsieh [50]	Suppress 0.5–2 Hz (eye blinks) and 43–59 Hz (high frequency disturbance)	Theta (4–7 Hz) amplitude exceeds mean amplitude of the eye-open baseline

^a^backslash (/) indicates no active suppression required for the user. The feedback threshold is usually designed based on the feedback condition of band activities

### Bias in BCI application to NF trials

This review applies Cochrane Risk of Bias Tool 2 (RoB2) [82] which is closely aligned with Methodological Expectations of Cochrane Intervention Reviews (MECIR) and The Cochrane Handbook [83]. We provided the risk of bias for the included studies related to the following domains: randomisation process(D1), deviations from intended interventions(D2), missing outcome data(D3), measurement of the outcome(D4), selection of the reported result(D5). RoB2 includes several domains, each assessed using signalling questions that are designed to be factual and facilitate judgements about the risk of bias. The response options for these signalling questions are: (1)Yes (2)Probably yes (3)Probably no (4)No (5)No information. Responses of “Yes” and “Probably yes” indicate a lower risk of bias, while “No” and “Probably no” suggest a higher risk of bias. If insufficient data are reported, we use the “No information” response. The RoB 2 tool maps the responses to signalling questions to a proposed risk-of-bias judgement for each domain D (see the full documentation [82] for details). The proposed judgement of each domain in response to the signalling questions are: (1)Low risk of bias: The study meets all criteria for low risk in this domain. (2)Some concerns: The study has some concerns in at least one domain. (3)High risk of bias: The study has a high risk of bias in at least one domain or some concerns in multiple domains

**Table 5:** A table to show percentage of risk of bias

Assignment to intervention (the ‘intention-to-treat’ effect), total number of studies = 14						
Risk level(%)	Randomisation process	Deviations from intended interventions	Missing outcome data	Measurement of the outcome	Selection of the reported result	Overall bias
Low risk	68.8	75	68.8	43.8	81.3	43.8
Some concerns	25	25	31.3	43.8	18.8	43.8
High risk	6.3	0	0	12.5	0	12.5

**Fig. 3:**
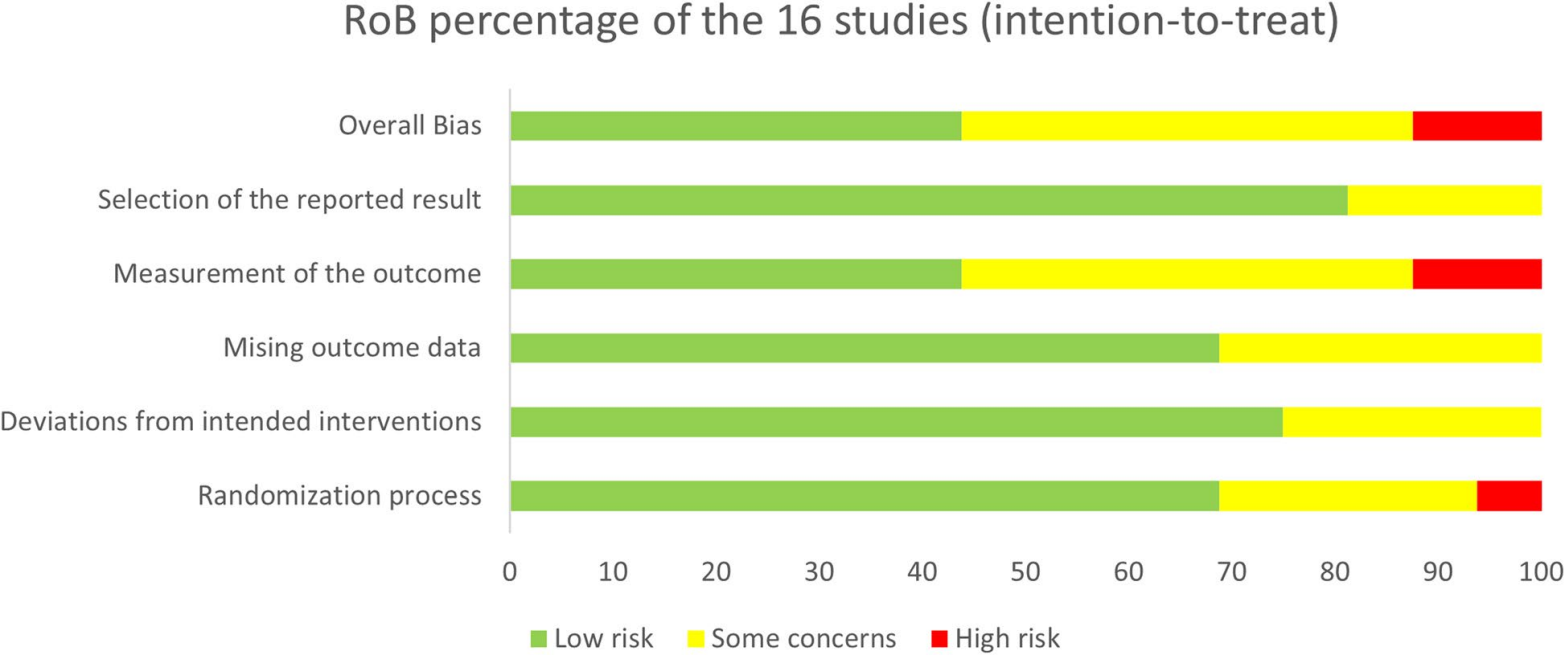
The bar chart shows risk of bias as percentage for the 16 included studies

**Fig. 4:**
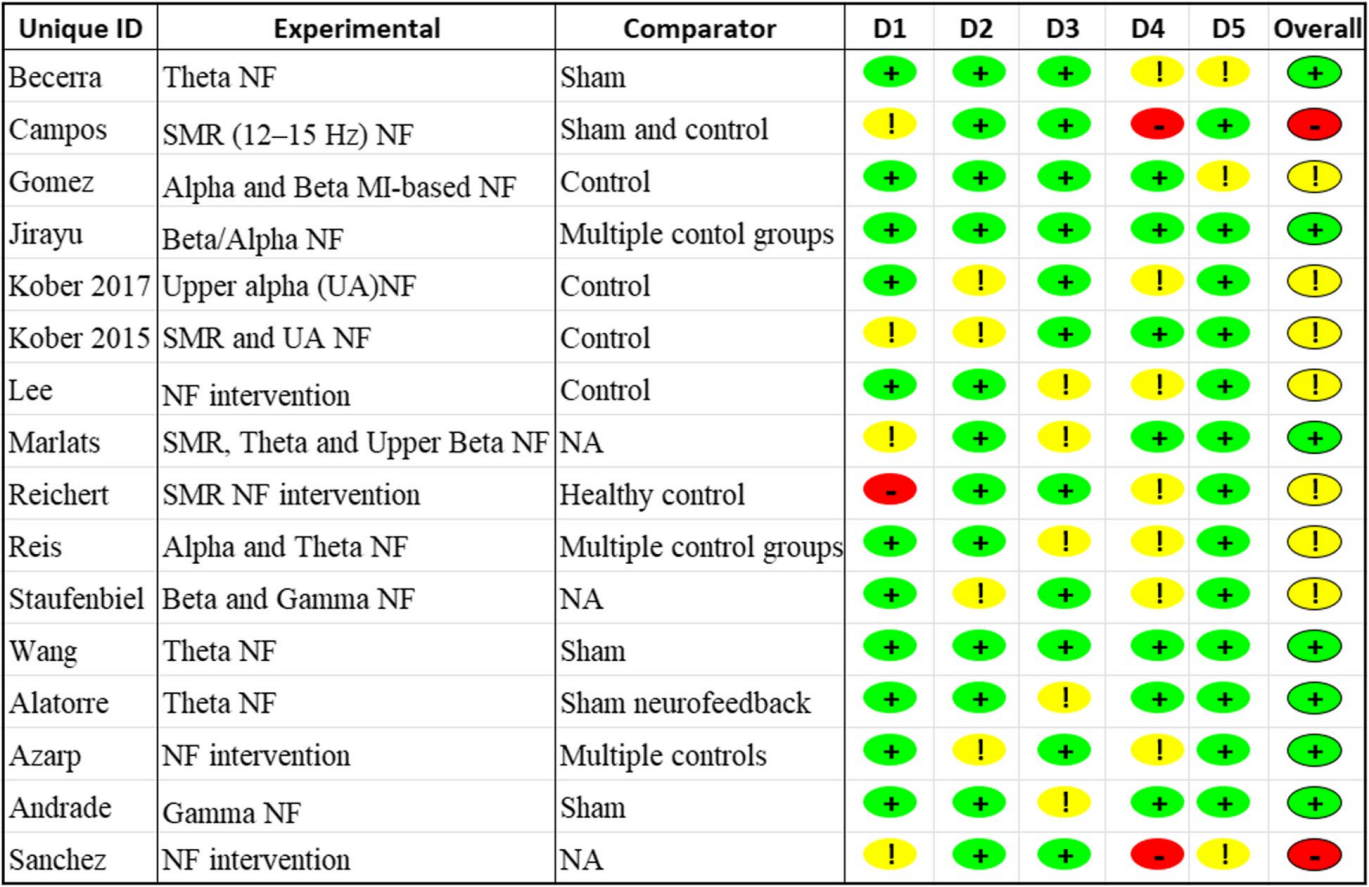
The figure shows the experimental design of treatment groups: experimental group and comparator group. Randomisation process(D1), deviations from intended interventions(D2), missing outcome data(D3), measurement of the outcome(D4), selection of the reported result(D5) indicate five aspects where the risk of bias can arise in a study. The risk signals are in green (low risk), yellow (some concerns), red (high risk). UA: upper alpha; NF: neurofeedback; SMR: sensorimotor rhythm; NA: not available

The overall bias for 16 studies was rated at three levels: 43.8% at low risk, 43.8% at some concerns and 12.5% at high risk (see Table 5, Figs. 3 and 4). The following sections present and challenges posed by the bias in BCI application to NF trials.

#### Randomisation process

There are concerns regarding potential sources of bias in participant selection and intervention assignment. Allowing the participants or the cognitive performance assessor to know the intervention assigned is not appropriate, since their knowledge of the assignment may affect outcome measurement. While some studies used rigorous designs, not all included control or sham groups, limiting the ability to make conclusive findings about the intervention’s true effects. The performed analysis indicates that blinding during the randomisation process were implemented to strengthen the validity of the results.

Six studies applied single-blind randomised controlled trials [49, 50, 54, 56, 60, 61], where five of them included sham groups in the experiment [50, 54, 56, 60, 61], one included a waitlist control group that received the same intervention after a waiting period[49]. Two study applied a double-blind randomised controlled trial [57, 58]. Three studies used a quasi-experimental design to compare the efficacy of neurofeedback training between healthy older people and stroke patients with memory deficits [51–53].

In Kober et al.’s experiment [51], sensorimotor rhythm (SMR) neurofeedback and up-regulation of upper alpha (UA) neurofeedback were assigned to two groups of subjects respectively: one with deficits in long-term memory performance and the other with deficits in their working memory performance. The study reported the intervention results separately but did not compare the effects of SMR and UA neurofeedback training on cognitive performance. The lack of direct comparison between the two training groups may limit the ability to make direct and definitive conclusions about the relative efficacy of these interventions.

Three studies out of the 16 included studies [48, 55, 57] did not apply any control or sham group as a comparator to the intervention group in their experimental design. However, parametric conditions were confirmed for the measured results for [55, 57], which reduced the impact of bias in estimation.

#### Deviations from intended intervention

Intended interventions may change over time. Gomez et al. [59] redesigned the protocol to include EEG analysis in the middle of the study. Only EEG data from participants recruited after the redesign were included in outcome measurements. The inconsistency of the trial results may have led to bias in the reported results. The protocol was then applied to an excluded study’s experiment[84] to analyse EEG-induced complexity by means of multi-scale entropy (MSE). The rest of the 15 studies did not mention any deviation from the intended intervention.

#### Missing outcome data

If the data for outcome were not available for all the assign-to-treat participants, then bias arises from the missing data. The data attrition could compromise the initial randomisation which influences the treatment effect estimate. Two studies addressed methods on how to handle missing data [49, 55]. Two studies mentioned participants dropping out without reporting techniques to impute missing data [47, 54]. In the absence of missing data handling, studies will suffer attrition bias before even conducting statistical analysis.

#### Measurement outcome: statistical aspects

In Reichert et al.’s study [52], intra-group analysis employed a paired-sample t-test for the EEG activities of each control participant (*N*=20), which gave an indication of the efficacy of NF training in the healthy controls. Non-parametric bootstrapping methods were employed to ascertain the degree of homogeneity in the control group. Since only one person received the intervention, single-case analysis was applied to compare and assess the training performance of the stroke patient with the control group. The application of single-case analysis requires the intervention and the control group to be in the same distribution, but the author did not ascertain if the two groups of people belonged to the same distribution.

For all the upper alpha and sensorimotor rhythm (SMR) based NF groups in Kober et al.’s article [51], regression analysis was carried out to reveal the EEG target power changes over the six training sessions. The healthy control groups’ normality (group sample size N<20) was not confirmed to meet parametric assumptions for a one-sample t-test, but the author applied a one-sample t-test to verify the consistency of the training effects on EEG signal powers. Similarly, a paired-sample t-test was carried out on the learning tasks scores of the healthy participants in the author’s later study [53] with a larger group sample size (N>20). A proper repeated-measures ANOVA on a healthy control’s resting EEG was applied to investigate EEG topographical distribution. The pre-post EEG power differences of a single stroke patient and healthy controls were analysed by single-case analysis.

Becerra et al. [60] found that the sample (*N*=7 for each group) distribution normality was not confirmed, so a non-parametric permutational ANOVA model was used to determine the association between groups and cognitive test results. A non-permutational t-test was performed for multiple comparisons of the results. As the number of comparisons increases, the intervention group and the control group are more likely to have at least one attribute difference due to selection bias. Multiple t-tests may then result in an inflation of the false positive rate. Jirayu et al. [46] applied two-tailed ANOVA to assess the continuous variables between the treatment groups, where each group had a sample size greater than 20. This number meets the sample size guideline. The author also used a repeated measurement generalised estimating equation (GEE) analysis to find out the effects of NF training in the separate treatment groups.

In contrast, Staufenbiel et al. and Campos et al.’s studies [56, 57] had small samples (N$\le$10) of participants in each intervention and control group, but neither the normality nor the homogeneity of variance was confirmed before running a parametric test for the outcome measures. This direct employment could lead to a tendency to overestimate or underestimate the NF effect. Sanchez-Cifo et al. [48] recruited 25 participants and did not confirm parametric conditions or conduct statistical comparisons data analysis. Instead, they focused on the application of machine learning models (e.g., SVM, Random Forest, Decision Tree) on real-time EEG for classifying emotional states. The study targeted practical effectiveness of the system in providing feedback, rather than testing for statistically significant changes.

Another study that demonstrated a lack of statistical consideration was by Wang and Hsieh [50]. They conducted between-subject ANOVAs to examine cognitive performance association with the intervention and sham group without claiming normality for the small-sample-size (N<10 for each group) data. The reliability of the computed test statistics may be compromised in this study. The chance of a false positive result may be increased in this case.

With a small sample size for each intervention, control and sham group (N<10), Reis et al. [54] performed comparisons between groups, testing for NF training effects and testing statistical independence between EEG measurements using non-parametric tests. With a small size (N<10) of participants in each group, instead of between-group analysis, Alatorre et al. [61] performed within-group analysis on pairs of time points to assess NF training effects using non-parametric tests. The false discovery rate (FDR) method was employed in their study to control a low portion of false positives, which avoids bias in measuring the outcome. The authors [54, 61], however, did not mention the assumption that the groups had the same spread. If the groups did not have the same spread, then a non-parametric test may give an invalid estimation the intervention effect.

In a later study, Gomez et al. [59] employed the Kolmogorov-Smirnov test to evaluate the normality of the pre- and post-training cognitive test scores distribution and used the Levene test to assess homoscedasticity before conducting comparison tests. This systematic approach ensured that data distribution and variance assumptions were carefully evaluated before proceeding with further analyses. When parametric assumptions were found to be violated, a non-parametric Mann-Whitney U-test was used to compare non-paired cognitive test scores between groups. Additionally, changes in each neuropsychological subtest and EEG power spectral density were also analysed using the Mann-Whitney U-test. To assess paired scores for pre- and post-training, the Wilcoxon signed-rank test was employed. Likewise, Lee et al. [49] estimated the differences between the intervention and control groups using Wilcoxon and Mann-Whitney tests. Since these are non-parametric methods, the lack of explicit reporting on data distribution assumptions is acceptable, as such methods inherently do not rely on parametric assumptions like normality. In a similar vein, comprehensive tests in Andrade et al. [58] included EEG processing with the Shapiro-Wilk test for normality. Non-parametric methods were used: the Mann-Whitney U-test for inter-group comparisons and the Wilcoxon signed-rank test for intra-group changes.

### EEG processing

Measuring EEG from the brain is useful because it reflects how the neurons in the brain network interact with each other. EEG recordings measured from the resting state are a sign of spontaneous neural activity, which can reveal the fundamental brain state [85]. Additionally, resting-state EEG recordings could be a useful tool to find out how NF training affects brain activities. Analysis of EEG activities in pre- and post-training conditions were taken into account before a conclusion was drawn about the efficacy of the NF training. In the included studies, some authors not only used EEG for generating feedback signals, but also recorded EEG obtained within the training session, or pre- versus post-training for analysis in outcome measures.

When dealing with off-line result analysis (both longitudinal (in time) and horizontal (group) comparisons), absolute power and relative power derived from spectrally analysed EEG were mostly used. EEG is the neural activity that underlies mental processes, and the pre-post changes in EEG represent the impact it has on brain function and behaviour. Four studies processed EEG for on-line NF training but did not report off-line EEG analysis [46–49]. Jirayu et al. [46] measured eye-open EEG to set up an attention level threshold for NF training tasks, but no further off-line EEG analysis. Similarly, Lee et al. [49] measured the participant’s incoming EEG data during a colour-word matching game and transformed it into an attention score, but the EEG data was not recorded for further analysis. The preliminary efficacy of NF was determined through cognitive assessments. In the absence of EEG off-line analysis, the objective information to evaluate the changes of before-after training in participants’ brain functions was limited.

#### Artefacts removal

EEG are sensitive recordings that are easily contaminated by cranial muscles even if the electrodes are positioned in the centre of the scalp. The artefact removal schemes hence were employed as one of the key processes of EEG signal processing. A significant challenge is to distil relevant data from the corrupted EEG signals and develop robust BCI that functions in a variety of situations.

In our review, Independent Component Analysis (ICA) was employed in the seven included studies [46, 48, 51, 54, 57, 60, 61] either on-line or off-line. Reis et al. [54] argued that ICA was not suitable when EEG data was from a low number (four) of active electrodes; on the other hand Jirayu et al. [46] employed ICA with Lifting Wavelet Transform (LWT) using four selected electrodes to enhance on-line artefacts removal efficiency on the data. In Kober et al.’s work [51], the eye blinks and muscle activities in the off-line EEG signals were rejected using a semi-automatic artefact rejection scheme with a rejection threshold for absolute voltage. The author also Z-transformed the power values to facilitate quantitative comparisons across frequency bands off-line. Likewise, artefact removal was done by rejecting epochs (within a window of 200ms) with voltage amplitudes exceeding a pre-set threshold in Staufenbiel et al. [57]’s off-line EEG analysis stage. Alatorre et al. [61] and Becerra et al. [60] computed the Z value of the absolute reward frequency band power and used it to adjust the active threshold. Although no specific artefact reduction methods were indicated in Reichert et al.’s study [52], on-line active suppression of eye-blink artefacts was employed during NF training (see Table 4). Artefact handling is more commonly conducted off-line by researchers. For example, Andrade et al. [58] excluded epochs with kurtosis values outside the range of 1 to 5 or peak-to-peak amplitudes exceeding 100 $\mu$V.

### Outcome measures

This section highlights the efficacy of BCI NF training from the included studies across various cognitive functions in both healthy and clinical populations, particularly older adults. These measures encompass a range of neuropsychological tests and EEG analyses that collectively show the impact of NF training on cognitive performance and brain activities.

Kober et al. [51] and Kober et al. [53] explored neurofeedback (NF) training’s impact on cognitive functions in stroke patients and healthy controls, using the California Verbal Learning Test (CVLT), Corsi Block Tapping Test (CBTT), and Visual and Verbal Memory Test (VVM). Kober et al. [53] also used the Test of Attentional Performance (TAP) and EEG resting measurements. In Kober et al. [51]’s study, stroke patients in the SMR group improved visuo-spatial short-term memory, verbal short- and long-term memory, and learning efficiency, while the Upper Alpha group improved verbal long-term memory. Healthy controls showed similar NF performance. Kober et al. [53] found significant memory improvements in stroke patients post Upper Alpha NF training, with EEG changes in delta and upper alpha power, underscoring NF’s potential for cognitive rehabilitation.

Similarly, Reichert et al. [52] assessed SMR NF training’s efficacy in mitigating sensorimotor interferences in stroke patients using neuropsychological tests and Event-Related Potentials (ERPs) recorded at the Pz electrode. Significant improvements in short- and long-term memory were observed in the NF group, with enhanced P3 amplitude in non-verbal learning tasks, paralleling cognitive gains seen in Kober et al. [51] and Kober et al. [53]. No significant changes were noted in the control group, emphasising NF’s specific benefits.

Likewise, Campos et al. [56] compared SMR NF training in healthy older adults to sham-NF (SNF) and no-NF (NNF) groups, using the Philadelphia Brief Assessment of Cognition (PBAC), Beck Anxiety Inventory (BAI), Beck Depression Inventory (BDI), and Delayed Matching to Sample Task (DMTS). The NF group showed significant improvements in DMTS performance and EEG theta, beta, and alpha power changes, particularly in the frontal and temporal regions. These EEG changes and working memory improvements are similar to those observed in Jirayu et al. [46] and Kober et al. [53].

In another study, Marlats et al. [55] observed significant cognitive improvements in elderly patients with MCI following SMR/theta NF training, using tools like the Montreal Cognitive Assessment (MoCA) and Rey Auditory Verbal Learning Test (RAVLT). Increased theta and alpha power post-training, particularly in frontal and central regions, were noted. These findings are comparable to those of Campos et al. [56] and Jirayu et al. [46]. However, the absence of a control group limits the conclusiveness of these findings.

In contrast, Becerra et al. [60] found significant cognitive enhancements in verbal processing, attention, and executive functions in the NF group compared to a sham-NF control group, assessed using WAIS-III and NEUROPSI. EEG analysis revealed significant reductions in theta and increases in alpha power, particularly in midline and left frontal leads, aligning with power changes in Campos et al. [56] and similar cognitive improvements in Kober et al. [51] and Kober et al. [53].

Additionally, Jirayu et al. [46] used the Cambridge Neuropsychological Test Automated Battery (CANTAB) to assess SWM and sustained attention, showing significant improvements in the intervention group. EEG analysis revealed changes in Beta/Alpha power ratios, indicating improved attention levels, similar to EEG power changes in Campos et al. [56] and Kober et al. [53]. The control group did not show significant improvements, underscoring the specific benefits of the NF training.

Azarpaikan and Torbati [47] investigated the effects of somatosensory and NF training on balance in older adults, using tools like the Biodex Balance System and Berg Balance Scale (BBS). Significant improvements in static and dynamic balance, fall risk reduction, and faster TUG times were noted in both training groups. NF training showed effectiveness in enhancing static balance, while somatosensory training improved dynamic balance. This study spotlights the potential of NF training to improve balance and reduce fall risk in the elderly, paralleling the cognitive and functional enhancements observed in the studies by Kober et al. [51], Kober et al. [53], and Becerra et al. [60].

Furthermore, Gomez et al. [59] assessed motor imagery-based NF training in elderly participants, finding significant improvements in visuo-spatial, oral language, memory, and intellectual functions using the Luria-AND test. EEG analysis showed increased relative power (RP) at 18 Hz and 21 Hz in the intervention group, particularly in frontal and temporal regions, similar to findings in Campos et al. [56] and Jirayu et al. [46]. The control group showed no significant changes.

On a different note, Staufenbiel et al. [57] investigated beta and gamma NF effects on memory and intelligence in the elderly, using placebo controls. Cognitive performance tests showed no significant improvements, despite increased beta and gamma activity, contrasting with cognitive gains in Kober et al. [51], Kober et al. [53], and Becerra et al. [60]. Andrade et al. [58] also employed gamma synchrony modulation and then evaluated its effects using neuropsychological tests using tools such as the Mini-Mental State Examination (MMSE), Free and Cued Selective Reminding Test (FCSRT), and Trail-Making Test (TMT). Another two EEG biomarkers, including Peak Alpha Frequency (PAF) and Theta/Beta Ratio (TBR), are used off-line to evaluate the effects. The results, in contrast, demonstrated improvements in episodic memory and executive functions in the NF group, while the sham feedback group showed no notable changes.

Moreover, Alatorre et al. [61] evaluated NFB’s impact on cognitive functions and EEG activity in older adults, finding significant improvements in executive functions and IQ compared to sham-NF controls. EEG analysis showed decreases in delta and theta activity and increases in beta activity post-training, similar to power changes in Campos et al. [56] and Becerra et al. [60]. These findings align with executive function enhancements in Jirayu et al. [46] and underscore NFB’s potential for long-term cognitive enhancement and brain activity modulation in aging populations.

Additionally, Reis et al. [54] compared NF, cognitive training (CT), and combined NFCT on working memory and cognitive control, using tasks like the Stroop and N-Back. The NF group showed significant improvements in working memory and cognitive control, with EEG analysis revealing increased alpha and theta power in the frontal and parietal lobes, aligning with findings from Campos et al. [56] and Kober et al. [53]. The CT group showed moderate cognitive improvements, while the NFCT group exhibited the most pronounced enhancements, including significant gains in working memory task performance and cognitive flexibility (the ability to switch between thinking about two different concepts or to think about multiple concepts simultaneously) as well as stronger neural responses during cognitive tasks. The effectiveness in modulating brain activities corroborates similar findings in Jirayu et al. [46] and Alatorre et al. [61].

Finally, Wang and Hsieh [50] employed Sham-Neurofeedback (Sham-NF) as a control group and assessed attention and working memory using the Attention Network Test (ANT) and Modified Sternberg Recognition Task, respectively. The study showed significant improvements in orienting and conflict scores on the ANT for both older and younger neurofeedback groups, while alerting scores did not increase. In the neurofeedback group, working memory function improved significantly post-training. EEG analysis revealed significant modulation of resting theta activity, particularly at the frontal-midline (Fz), with increased theta amplitude during training sessions, indicating effective neurofeedback. These findings align with enhanced working memory and attention improvements observed in Reis et al. [54], Jirayu et al. [46], and Becerra et al. [60], and with increased theta activity as seen in Campos et al. [56] and increased upper alpha activity observed in Kober et al. [53]. The study shows the efficacy of frontal-midline theta uptraining for cognitive aging interventions.

In summary, these findings stress the potential of NF interventions to enhance memory, attention, executive functions, and balance, particularly through targeted electrode placements and optimised training protocols. The consistent improvements across different cognitive domains and EEG power changes highlight the importance of continued research and refinement of NF methodologies to maximise their efficacy and applicability in cognitive rehabilitation for aging populations.

## Discussion

The investigation into the application of BCI and NF technologies in older adults reveals critical insights and underscores significant challenges, particularly in older adults. This review examines the relationship between system design, feedback electrode placement, bias from experimental design, bias from statistical analysis and the observed outcomes in 16 studies

System design and electrode placement are crucial factors influencing the efficacy of BCI and NF interventions. Most studies utilised research-grade EEG systems like the NeXus-10 MKII and Brain Products amplifiers, known for their precision and robustness against noise. Such systems are essential for tasks requiring accurate classification of EEG patterns associated with specific cognitive processes, particularly in older adults whose EEG signals are more susceptible to noise and interference.

The placement of electrodes, typically following the International 10–20 system, directly impacts the training outcomes. For instance, studies targeting memory improvements often placed electrodes over the central and parietal regions, while those focusing on attention used frontal placements. This strategic placement aligns with the brain regions responsible for these functions, enhancing the specificity and effectiveness of the interventions. The importance of this approach is reflected in the studies by Kober et al. [51] and Kober et al. [53], which showed significant improvements in memory and attention through targeted neurofeedback protocols.

A major concern in the 16 studies was the randomisation process. Many studies lacked adequate control or sham groups, making it difficult to attribute improvements solely to the NF intervention. The absence of blinding during the randomisation process in some studies further compromised result validity. For instance, Marlats et al. [55], Sanchez-Cifo et al. [48] and Staufenbiel et al. [57] did not include control groups, which raises concerns about whether observed cognitive improvements were due to the intervention or other confounding factors. Well-designed control groups are essential for attributing improvements to the intervention itself, and without them, it’s challenging to distinguish between true intervention effects and placebo effects.

Additionally, studies often assigned training protocols based on participants’ health conditions, creating unequal distribution of confounding factors that could affect outcomes. This approach necessitates the use of stratification in small trials for meaningful subgroup analysis. However, well-controlled studies with larger samples of older adults are still lacking. Non-randomised studies, while addressing population differences, may lead to misleading comparisons about treatment outcomes. Control groups must be matched in demographics and cognitive behaviours to ensure comparability.

For statistical aspects of RoB, a rigorous trial should consider appropriate statistical methods to quantify or eliminate outcome measurement bias. Measurement bias can result in associations between variables that are either larger or smaller than the actual association [86]. Typically, cognitive performance is gauged using neurophysiological test scores to enable statistical evaluations. Importantly, the size of the sample can significantly affect statistical assessments, especially when decoding accuracy is considered. Importantly, the size of the sample can significantly affect statistical assessments, especially when decoding accuracy is considered. For instance, with small sample sizes below 100 observations, decoding accuracy must be substantially above chance levels to be deemed statistically significant [87]. Therefore, careful consideration should be given to the size of the sample and the ensuing implications for decoding accuracy thresholds when conducting parametric analyses.

According to the analysis, many studies used parametric analysis methods while using a small sample size without checking if the sample population violated the parametric normality assumptions.If the sample size satisfies the guideline for non-normal data that a certain number of samples should be reached, then a parametric test can be performed on the data [88]. There is a need to perform adequate statistical tests on the outcome to better understand the effects of NF intervention and hence the BCI application.

The outcome measures varied across studies but consistently highlighted the benefits of NF training on specific cognitive functions. For instance, the significant improvements in visuo-spatial short-term memory and verbal memory in stroke patients reported by Kober et al. [51] and the enhanced working memory and cognitive control in older adults observed by Reis et al. [54] emphasise the potential of NF training. EEG analyses across studies consistently showed changes in power within specific frequency bands, such as increased alpha and theta power in the frontal and parietal lobes, as noted in Campos et al. [56] and Jirayu et al. [46]. These changes suggest enhanced neural efficiency and cognitive function.

There were some protocols targeting on the same frequency-band modulation but yields contrast results. This difference between the studies highlights the importance of optimised NF protocols. For example, in Staufenbiel et al. [57]’s study, the absence of cognitive improvements despite neural activity changes suggests that the training parameters, such as feedback thresholds, targeted biomarkers, or the duration of training, may not have been effectively tuned to drive cognitive benefits. On the other hand, Andrade et al. [58]’s study used carefully calibrated feedback based on gamma synchrony and included additional biomarkers (PAF and TBR) for comprehensive analysis, which results in in measurable cognitive improvements. These findings emphasise the critical role of optimising NF protocols, including the selection of appropriate EEG biomarkers, adaptive thresholds, and training strategies. By this, ones can ensure that neural changes are accurately targeted and translated into measurable cognitive improvements.

### Significance

The significant findings from these studies highlight the potential of BCI and NF technologies to enhance specific cognitive functions in older adults, such as working memory, attention, and executive functions. Reis et al. [54] showed significant improvements in working memory and cognitive control with increased alpha and theta power in the frontal and parietal lobes, aligning with findings from Campos et al. [56] and Kober et al. [53]. The detailed analysis of system design and electrode placement provides valuable insights for optimising NF interventions. For example, Kober et al. [53], Kober et al. [51] and Campos et al. [56] showed that strategic electrode placement and precise EEG recording can lead to significant cognitive and neural improvements, emphasising the need for tailored NF protocols. However, the presence of bias and the need for more rigorous experimental designs pose challenges that must be addressed in future research. The variability in system design, electrode placement, and statistical methodologies across studies indicates a need for standardised protocols to ensure comparability and reproducibility of results.

### Challenges and applications of BCI in older adults

Several challenges remain in applying BCI technology to older adults. The variability in NF training protocols and system designs across studies highlights the need for standardised methods to ensure consistent results. Also, the need for user-friendly interfaces and the requirement for robust statistical analyses must be overcome. The findings suggests the importance of user-friendly NF paradigms to ensure high acceptance and adherence among older adults. This necessitates well-designed trials to minimise bias from experimental design. Additionally, the susceptibility of older adults’ EEG signals to noise and interference, as noted by [89], requires further refinement of BCI systems to enhance signal quality and user experience.

BCI systems designed for older adults must address their unique cognitive and physical limitations. Older adults might find it difficult to engage with complex NF protocols or MI tasks, which could impact the efficacy of the training. The need for adaptive NF training paradigms that cater to the unique neural and cognitive profiles of older adults is crucial. This requires further work on online EEG processing to generate personalized feedback stimuli for each user. Furthermore, understanding the performance gaps between older and younger individuals in BCI tasks is essential to enhance BCI performance. Research has revealed altered sensorimotor networks in older adults during visuo motor tracking tasks [90]. Chen et al. [89] also found decreased cortical lateralisation and reduced EEG power in older subjects, resulting in lower BCI classification accuracy. The findings suggest that age-related differences in functional brain activity must be considered when developing BCI and NF systems for older populations. Also, Older adults are less affected by cognitive fatigue during MI tasks, but their classification accuracy is lower compared to younger participants [91]. This indicates the need for appropriate algorithms, such as the deep learning method that Li et al. [91] found to be effective, to improve BCI performance in older adults. Noise in EEG signals may also contribute to the lower MI classification accuracy in older adults, as effective artefact removal is crucial for accurate brain activity interpretation. To address this, deep learning models such as IC-U-Net [92] and ART [93] offer advanced solutions for denoising multi-channel EEG signals. IC-U-Net’s U-Net architecture with ensemble loss functions effectively suppresses various noise types, while ART’s transformer-based design captures complex temporal and spatial dependencies in EEG. Incorporating these models into BCI systems could help bridge noise-related performance gaps.

To address the unique needs of older users, BCI development should incorporate user feedback through rating scales such as the System Usability Scale (SUS) and qualitative methods like interviews and focus groups. These approaches can help design more user-friendly and effective BCI systems. A notable finding among the included studies is that only two [46, 48] employed lightweight EEG devices for neurofeedback training. However, neither study conducted off-line EEG analysis, despite claiming efficacy in cognitive enhancement. The lack of post-training evaluations, such as EEG biomarker analysis, raises concerns about the robustness of their efficacy assessments. Off-line analysis refines algorithms and identifies patterns of biomarkers to improve real-time feedback accuracy. This highlights the need for research into portable BCI systems for older adults. Off-line analysis can help validate reliability of systems tailored to older adults.

As a result, the current body of research supports the potential of NF training to improve specific cognitive functions and modulate brain activity in older adults. However, addressing methodological concerns, refining system designs, and optimising electrode placement are critical for maximising the efficacy of NF interventions. Future research should focus on standardising NF protocols, enhancing BCI system usability based on specific needs of older adults, addressing age-related changes in EEG, and exploring long-term benefits to solidify NF training’s role in cognitive rehabilitation for the aging population. These efforts will ensure more reliable and impactful outcomes, addressing both the technical and methodological challenges identified in this systematic review.

## Conclusion

The systematic review follows PRISMA guidelines. It extracted and analysed relevant studies (N = 16) on BCI and NF applications in older adults. Despite certain biases in randomisation processes, intervention changes, handling of missing outcome data, and statistical analyses, the studies provide valuable insights and highlight areas for future research.

Research-grade BCI systems, known for their robustness against noise, are crucial for tasks that require precise EEG data classification, especially for older adults who are more susceptible to EEG signal interference. The review indicates that memory and attention are the cognitive domains most positively impacted by NF training in older adults.

Future research should aim to construct randomised controlled trials with larger sample sizes, appropriate control groups, and rigorous blinding to better evaluate the efficacy and feasibility of NF as a rehabilitation tool for older adults. Understanding the performance differences and neural-cognitive mechanisms in this population can help adapt BCI systems to their specific brain dynamics, potentially using BCI as a preventive measure against cognitive impairment.

New opportunities exist for improving experimental design, NF training paradigms, EEG processing, and portable BCI system design. With more focused studies on cognitive improvement in healthy and mildly cognitively impaired older adults, the field of EEG-based BCI applications can continue to grow, providing more abundant and reliable data for enhancing cognitive functions in this population.

## Data Availability

All information found within this review have been taken from previously published data.

## Appendix

**Table 6:** The table shows EEG recording apparatus and EEG processing software

Reference	EEG apparatus			Software(Bold) and processing	
	EEG Cap	On-line	Off-line	On-line	Off-line
Alatorre et al. [61]	ElectroCap™ (International Inc.; Eaton, Ohio)	MEDICID IV System. Sampling rate at 200 Hz.	MEDICID IV System. Sampling rate at 200 Hz	**Track Walker:** Theta absolute power was segmented into a 1280ms-window, displacing 20ms each time. z-value for theta AP was computed	**A custom software** [94, 95]: statistical nonparametric mapping(SnPM) was performed. Artefact-free segments of 2.56s were selected. Cross-spectral matrices and absolute EEG power was computed by fast Fourier Transform (FFT). Relative power of interest was Z-transformed for NF
Andrade et al. [58]	20-channel cap	Neuroelectrics, Barcelona, Spain; Sampling rate at 500 Hz	NIC^®^ EEG amplifier, Neuroelectrics	**MATLAB Psychtoolbox v3 and System Level Simulations (SLS):** no method reported	**MATLAB Brainstorm: ** gamma, peak alpha and theta/beta ratio were computed using Welch’s PSD estimation on 1 s-window with 25% overlapping in each 2-seconds epoch
Azarpaikan and Torbati [47]	20-channel cap	Flex Comp Infiniti encoder and TT-USB interface unit. Sampling rate at 256 Hz	Flex Comp Infiniti encoder. Sampling rate at 256 Hz	**BioGraph Infiniti Software System:** no method reported	**Brain Vision Analyzer: ** Absolute SMR and theta power were elicited by complex demodulation
Becerra et al. [60]	19-lead cap	MEDICID IV System	MEDICID IV System	**MEDICID IV:** Band power was computed by FFT. Data was Z-transformed. 20 ms of absolute band power was computed every 5 ms for feedback	**MEDICID IV:** Absolute and relative power of feedback frequencies were computed by FFT for analysis
Campos et al. [56]	19-channels, (Waveguard connect)	ProComp Infiniti differential amplifier (Thought Technology Ltd)	NeuronSpectrum-4/EP system	**BioGraph Infiniti software:** Spectral amplitudes were computed on raw 1s EEG segments	**MATLAB EEGLAB toolbox:** EEG data were digitised into non-overlapping epochs time-locked to the task condition. The eye muscle artefacts were rejected by independent component analysis (ICA)
Gomez et al. [59]	8-lead elastic cap	g.USBamp amplifier (Guger Technologies). Sampling rate at 256 Hz.	g.USBamp amplifier (Guger Technologies). Sampling rate at 256 Hz.	**BCI2000:** Spatial nearest - neighbour Laplacian was applied over feedback electrodes.	**Matlab R2011b:** The artefacts were rejected using ICA. Band power spectral density was computed using nonparametric Welch method with 32s Hamming window.
Jirayu et al. [46]	14-channel Emotiv cap	Emotiv EPOC headset. Sampling rate at 128 Hz	Emotiv EPOC headset. Sampling rate at 128 Hz	**MATLAB:** ICA with Lifting Wavelet transform (LWT) to improve artefact removal efficiency. Power spectrum was computed by FFT with a Hanning Window sized 256 samples. Moving average of attention (ratio of Beta/Alpha) value in the window of 2 seconds was calculated. Each processed data block size is assigned to 64 samples	No processing software or method reported
Kober et al. [53]	10-channel cap	NeXus-10 MKII, Mind Media BV. Sampling rate at 256 Hz	Not clear	**BioTrace+(Mind Media):** Power spectra was computed using FFT, with 10$\%$ Hannning window	**Brain Vision Analyzer:** Frequency powers were calculated using the built-in method of complex demodulation. The data was Z-transformed. All epochs with artefacts were excluded from the analysis
Kober et al. [51]	10-channel cap	NeXus-10 MKII, Mind Media BV. Sampling rate at 256 Hz	Not reported	**BioTrace+(Mind Media):** Power spectra was computed using FFT, with 10$\%$ Hannning window	**Brain Vision Analyzer:** Frequency powers were calculated using the built-in method of complex demodulation. The data was Z-transformed. All epochs with artefacts were excluded from the analysis
Lee et al. [49]	Self-developed EEG headband with 2 dry electrodes	Recording system not reported	Off-line EEG not recorded	**Custom platform:** Spatial pattern filtering computed a model for discriminating attentive and inattentive states. A classifier in the model transformed the patterns into attention level for NF. EEG data was quantified into attention score at every 200 ms	No processing software or method reported
Marlats et al. [55]	Electro Cap (International Inc. Elmiko Medical)	EEG Digitrack Biofeedback plus module (Inc Elmiko Medical). Sampling rate at 512Hz	EEGDigitrack SimplEEG32. Sampling rate at 500 Hz	**EEG Digitrack Biofeedback plus module (Inc Elmiko Medical):** Moving averaged EEG rhythms (delta, theta, alpha, SMR, and lower beta) were computed	**Python and R:** ICA was used to reject artefacts and the recorded EEG passed a rejection test with a covariance-based approach. Relative band power was computed by FFT and averaged with Welch method
Reichert et al. [52]	10-channel cap	10-channel system NeXus-10 MKII, Mind Media. Sampling rate at 256 Hz	16-channel g.USBamp (g.tec). Sampling rate at 500 Hz	**Brain Vision Analyzer(Brain Products GmbH):** Band power was computed using built-in complex demodulation	**Brain Vision Analyzer software (Brain Products GmbH):** The data was Z-transformed. All epochs with artefacts were excluded from the analysis
Reis et al. [54]	actiCAP and EASYcCAP (Brain Product)	QuickAmp (Brain Products) or ActiCHamp (Brain Products). Sampling rate at 500 Hz	QuickAmp (Brain Products) or ActiCHamp (Brain Products). Sampling rate at 500 Hz	**Custom platform in BCI++ platform (developed by C++ and Matlab):** No artefact rejection was performed. Relative power of interest was calculated within a 1s-window and update visually to the user every 200ms	**EEGLAB by MATLAB and OriginLab:** Two-way least square FIR filter was applied to discard muscle artefacts and equipment noise. Ocular artefacts were removed based on ICA. Frequency bands absolute and relative PSD were computed
Sanchez-Cifo et al. [48]	2-channel EEG device Muse 2	Muse 2. Sampling rate at 256 Hz	/	**Python and MATLAB:** Frequency bands were computed to train Support Vector Machine (SVM) for emotional state classification	No processing software or method reported
Staufenbiel et al. [57]	EEG cap (g.tec medical engineering GmbH)	USB Biosignal Amplifier (g.tec medical engineering GmbH). Sampling rate at 256 Hz	USB Biosignal Amplifier (g.tec medical engineering GmbH). Sampling rate at 256 Hz	**Custom-built software:** Band power was computed by FFT with 1-second delay. Band power level was based on a moving average of 30s updated continuously. Average power was calculated over epochs per second	**Brain Vision Analyzer 2.0 (BrainProducts):** Data was subjected to ocular correction. All epochs (200ms-window) with artefacts were rejected
Wang and Hsieh [50]	Neuroscan Q-cap AgCl-32 electrode cap	ProComp Infiniti differential amp and software. Sampling rate at 256 Hz	Synamps2 (Neuroscan). Sampling rate at 500Hz	**BioGraph Infiniti software:** IIR filter was applied to extract frequency-specific bands. EEG signals were segmented into 1 Hz with 1-second time window. Reward EEG frequency bands were extracted through FFT	**Scan 4.3 acquisition software (Neuroscan):** To acquire artefact-free data, ocular artefacts reduction method was employed. Absolute EEG power was computed by FFT

## Abbreviations

EEG: Electroencephalogram
NF: Neurofeedback
MCI: Mild cognitive impairment
BCI: Brain-computer-interface
ADHD: Attention deficit hyperactivity disorder
SLR: Systematic Literature Review
PICO: Patient/population, intervention, comparison and outcomes
TMS: Transcranial magnetic stimulation
PRISMA: Systematic Reviews and Meta-Analyses
RoB2: Cochrane Risk of Bias Tool 2
MECIR: Methodological Expectations of Cochrane Intervention Reviews
MSE: Means of multiscale entropy
ANOVA: Analysis of Variance
SMR: Sensorimoter rhythm
GEE: Generalised estimating equation
FDR: False discovery rate
NFT: Neurofeedback cognitive training
CAU: Care as usual tratment
MI: Motor imagery
ERD: Event-related desynchronisation
ERS: Event-related synchronisation
UA: Upper Alpha
ICA: Independent Component Analysis
LWT: Lifting Wavelet Transform
RP: Relative power
